# A European survey on the practice of nutritional interventions in head–neck cancer patients undergoing curative treatment with radio(chemo)therapy

**DOI:** 10.1007/s00405-021-06920-4

**Published:** 2021-06-19

**Authors:** Federico Bozzetti, Cristina Gurizzan, Simon Lal, Andre’ Van Gossum, Geert Wanten, Wojciech Golusinski, Sefik Hosal, Paolo Bossi

**Affiliations:** 1 Faculty of Medicine, University of Milan, Milano, Italy; 2 Medical Oncology, Department of Medical and Surgical Specialties, Radiological Sciences and Public, Health University of Brescia, ASST-Spedali Civili, Brescia, Italy; 3 University of Manchester, Manchester, UK; 4 Intestinal Failure Unit, Salford Royal NHS Foundation Trust, Salford, UK; 5 Clinic for Intestinal Diseases and Nutritional Support Gastroenterology Service, Erasme Hospital, Brussels, Belgium; 6 Intestinal Failure Unit, Department of Gastroenterology and Hepatology, Radboud University Medical Centre, Nijmegen, The Netherlands; 7 Department of Head and Neck Surgery, The Greater Poland Cancer Centre, Poznan University of Medical Sciences, Poznań, Poland; 8 Department of Otolaryngology - Head and Neck Surgery, Faculty of Medicine, Atilim University, Ankara, Turkey

**Keywords:** Survey, Nutritional support, Head and neck cancer, Tube feeding, Curative treatment

## Abstract

**Purpose:**

As the practice of nutritional support in patients with head and neck cancer (HNC) during curative radio(chemo)therapy is quite heterogeneous, we carried out a survey among European specialists.

**Methods:**

A 19-item questionnaire was drawn up and disseminated via the web by European scientific societies involved in HNC and nutrition.

**Results:**

Among 220 responses, the first choice was always for the enteral route; naso-enteral tube feeding was preferred to gastrostomy in the short term, while the opposite for period longer than 1 month. Indications were not solely related to the patient’s nutritional status, but also to the potential burden of the therapy.

**Conclusion:**

European HNC specialists contextualize the use of the nutritional support in a comprehensive plan of therapy. There is still uncertainty relating to the role of naso-enteral feeding versus gastrostomy feeding in patients requiring < 1 month nutritional support, an issue that should be further investigated.

## Introduction

It is well-known that patients with head–neck cancer (HNC) represent a significant proportion of the cancer population with high rates of malnutrition evaluated both as prevalence and severity of weight loss [1]. This is due to the combined effect of reduced food intake because of chewing and swallowing problems associated with tumour localization, chronic poor dietary habits and tumour-related inflammation [2]. Furthermore, malnutrition may be aggravated by adverse effects of anti-cancer treatments on patient’s oral cavity and pharyngeal mucosa, as well as by the chemotherapy-induced sarcopenia [3].

The resultant malnutrition leads to reduced tolerance and response to oncologic therapies, shorter survival and poorer quality of life (QoL) in the general oncologic population as well as, specifically, in HNC patients [4].

Both the European Society of Parenteral and Enteral Nutrition (ESPEN) [5] and the American Society of Parenteral and Enteral Nutrition [6] recommend nutritional support in these patients which should be administered preferentially by enteral route or, as a second choice, intravenously, if the gut is not accessible or working. Recently, a mixed approach of oral nutrition combined with supplemental parenteral nutrition has gained wide acceptance in patients who are moderately hypophagic [7, 8].

The purpose of this paper is to report the results of a recent European survey addressed to clinicians involved in the care of HNC patients undergoing radio(chemo)therapy with primary curative or postoperative intent. Main aims of the survey were to define the most common approaches to nutritional support and to consider the role of further dialogue between oncologists and nutritionists, to clarify any grey areas that exist in the nutritional approach adopted by clinicians to optimize the modality of nutritional interventions.

## Materials and methods

The questionnaire, shown in Table 1, was posted as a web-survey link in the ESPEN and EHNS (European Head and Neck Society) websites from May to September 2019. The members of the two societies were alerted about this survey by e-mail.

**Table 1:** Contents of the questionnaire

Q1	Date of compilation (_____)
Q2	Specialty Medical oncology Radiation oncology Head and neck surgeon Nutritionist Dietist Internist Other (specify)
Q3	Type of institution Community hospital Academic centre Cancer centre Other (specify)
Q4	Country (_____)
Q5	Number of patients/year with Head and Neck cancer receiving postoperative or curative RT alone or combined RT + systemic therapy, who are treated in your Centre/Institution? (_____)
Q6	Is a Nutrition Service/Unit available in your Centre/Institution? Yes No
Q7	Is a Radiologic/Endoscopic expertise available to insert a gastrostomy in your Centre/Institution? Yes No
Q8	Do you routinely use a validated nutrition screening tool (e.g. MUST, MNA, MST, NRS 2002, other) to identify malnutrition risk in patients undergoing radio(chemo)therapy for patients with head and neck cancer? Yes No
Q9	If you expect that your patient will become severely dysphagic during a course of RT and/or CT, and a dietary counselling (including use of oral supplements) is not feasible or sufficient, do you start in hospital with the so called “prophylactic nutritional support” that is, as soon as possible ? Yes No
Q10	If Q9 yes, what do you use*?* *More than 1 answer is possible, please score your preference from 1 (higher preference) to 5 (lower preference)* Nasogastric/jejunal tube feeding (_____) Feeding tube directly entering into the stomach/jejunum (PG/PJ) (_____) Intravenous feeding through a cannula introduced in a peripheral vein and reaching the cava vein (_____) Intravenous feeding through a cannula inserted in a central (subclavicular or in the neck) vein (_____) Other (specify) (_____)
Q11	Which of the following criteria would you follow to start an enteral or a parenteral or a mixed enteral/parenteral prophylactic nutritional support? *More than 1 answer is possible, please score your preference from 1 (higher preference) to 6 (lower preference)* Planned radiation dose on oral and oropharyngeal mucosa and/or pharyngeal constrictor muscles (_____) Use of concurrent systemic therapy (_____) Altered nutritional or inflammatory parameters (ie: weight loss, Glasgow prognostic score, low phase angle, sarcopenia) before/during therapy (_____) Postoperative vs curative setting of radiotherapy (_____) Multiparameter scores predicting the need of GI-tube (_____) Other (specify) (_____)
Q12	If your patient needs a nutritional support at home for a period ranging from 2 weeks to 1 month because he/she cannot eat by mouth, which procedure would you use? *Please score your preference from 1 (higher preference) to 5 (lower preference)* Nasogastric/jejunal tube feeding (_____) Feeding tube directly entering into the stomach/jejunum (PG/PJ) (_____) Intravenous feeding through a cannula introduced in a peripheral vein and reaching the cava vein (_____) Intravenous feeding through a cannula inserted in a central (subclavicular or in the neck) vein (_____) Other (specify) (_____)
Q13	Please specify the reasons for your choice to Q12 *More than 1 answer is possible, please score your reason from 1 (first, main reason) to 6 (lower reason*) Cost (_____) Safety (_____) Comfort (_____) Easy to use (_____) Efficacy (_____) Other (_____)
Q14	If your patient needs a nutritional support at home for a period probably longer than 1 month because he/she cannot eat by mouth, which procedure do you use? *Please score your preference from 1 (higher preference) to 5 (lower preference)* Nasogastric/jejunal tube feeding (_____) Feeding tube directly entering into the stomach/jejunum (PG/PJ) (_____) Intravenous feeding through a cannula introduced in a peripheral vein and reaching the cava vein (_____) Intravenous feeding through a cannula inserted in a central (subclavicular or in the neck) vein (_____) Other (specify) (_____)
Q15	Please specify the reasons for your choice to Q14 *More than 1 answer is possible, please score your reason from 1 (first, main reason) to 6 (lower reason*) Cost (_____) Safety (_____) Comfort (_____) Easy to use (_____) Efficacy (_____) Other (_____)
Q16	Do you ask your patients about his/her preferences when more nutritional approaches are possible? Yes No
Q17	If yes (Q16), which is, according to your experience, their more common preference about the way to be fed when patients are almost/totally aphagic? *More than 1 answer is possible*, *please score your preference from 1 (higher preference) to 5 (lower preference)* Nasogastric/jejunal tube feeding (_____) Feeding tube directly entering into the stomach/jejunum (PG/PJ) (_____) Intravenous feeding through a cannula introduced in a peripheral vein and reaching the cava vein (_____) Intravenous feeding through a cannula inserted in a central (subclavicular or in the neck) vein (_____) Indifferent (specify) (_____)
Q18	Do you consider advanced age as a variable to be considered in suggesting an earlier nutritional support? Yes No
Q19	In case, which is the age cut-off do you generally consider as at higher risk of malnutrition, therefore, prompting prophylactic nutritional support? > 65 > 70 > 75 > 80

*CT* chemotherapy, *RT* radiotherapy, *PG* percutaneous gastrostomy, *PJ* percutaneous jejunostomy

The survey consisted of 19 items designed to investigate approaches to the nutritional management of HNC patients undergoing treatment with radio(chemo)therapy.

Questions concerned clinicians’ choices regarding the need for (supplemental or total) enteral, parenteral or mixed enteral–parenteral nutrition in the context of severe hypophagia both for a period ranging from 2 weeks to 1 month and more than 1 month in HNC patients while undergoing curative and/or postoperative radiation therapy (RT), with or without concurrent systemic chemotherapy.

We evaluated clinicians’ preferences for “prophylactic nutritional support”: nasogastric/jejunal tube feeding, feeding tube directly entering into the stomach/jejunum (PG, percutaneous gastrostomy or PJ, jejunostomy), intravenous feeding through a cannula introduced in a peripheral vein, intravenous feeding through a cannula inserted in a central vein or other options according to clinicians. The reasons for choosing different types of nutritional support were investigated: cost, safety, comfort, easy to use, efficacy or other.

We also explored if any validated nutrition screening tools (e.g. MUST, MNA, MST, NRS 2002 as summarized by Reber and colleagues [9], other) were routinely used to identify malnutrition risk and which criteria guided the choice to commence prophylactic nutrition.

Moreover, we asked clinicians to comment on patients’ preferences for nutritional support and whether the patient’s age was considered.

Responses to the survey were analysed and reported in a descriptive manner.

## Results

### Collection of the questionnaire

There were 220 responses to the questionnaire. As depicted in Table 2, the responders included head and neck surgeons (25%), nutritionists (19%) and dieticians (17%), radiation oncologists (15%) and medical oncologists (5%). These specialists reported that they treated a median of 100 patients/year with postoperative or curative RT or concurrent RT-systemic therapy and belonged in 36% of cases to community hospitals, with 27% of respondents working in academic hospitals and 27% in Cancer Centres. More than 90% respondents reported having a nutrition service and radiologic/endoscopic expertise available in their institutions. Countries contributing more than 5% to the survey included: Italy (19%), United Kingdom (17%), Belgium (11%), Spain (11%), Netherlands (5%), Sweden (5%) and Czech Republic (5%) (Table 2).

**Table 2:** Specialty, institutional services available and countries of respondents contributing to the survey

	*N* (%)
Specialty	
Medical oncology	10 (5)
Radiation oncology	34 (15)
Head and neck surgeon	55 (25)
Nutritionist	42 (19)
Dietist	37 (17)
Internal medicine specialist	8 (4)
Other specialties	34 (15)
Type of institution	
Community hospital	79 (36)
Academic hospital	59 (27)
Cancer centre	60 (27)
Other	22 (10)
Availability of nutrition service/unit	
Yes	205 (93)
No	15 (7)
Availability of radiologic/endoscopic expertise	
Yes	206 (94)
No	14 (6)
Country contributed ≥ 5%	
Italy	41 (19)
Belgium	24 (11)
Netherlands	11 (5)
Spain	25 (11)
Sweden	10 (5)
United Kingdom	37 (17)
Czech Republic	11 (5)
Country contributed 1–4.9%	
Austria	3 (1)
Finland	2 (1)
Greece	5 (2)
Hungary	9 (4)
Ireland	2 (1)
Poland	2 (1)
Portugal	2 (1)
Switzerland	8 (4)
Turkey	8 (4)
Croatia	2 (1)
Belarus	2 (1)
Lithuania	2 (1)

Denmark, Germany, Romania, Ecuador, India, Thailand, Cyprus, Indonesia, Australia, Chile, Egypt, China, USA, Norway Country contributed < 1% to the survey

### Use of prophylactic nutritional support

**Fig. 1:**
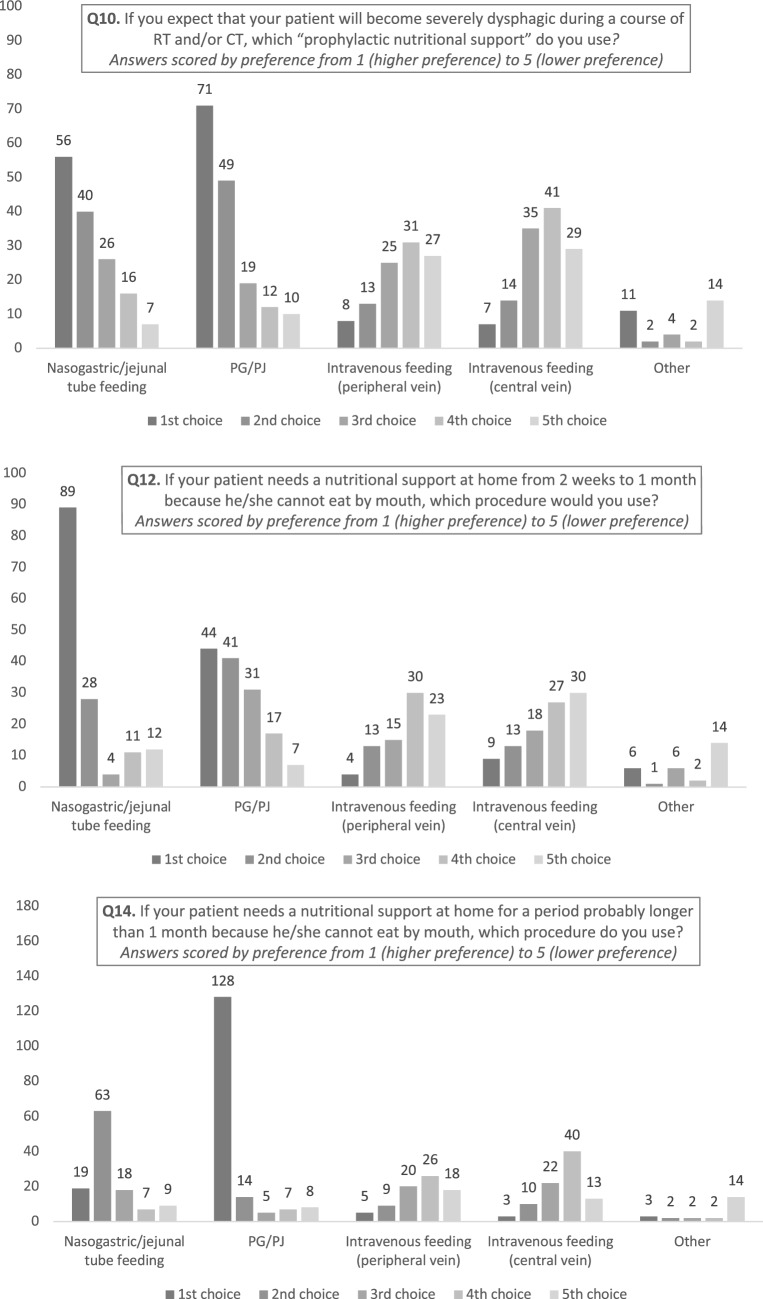
**a** Histograms representing survey responses to question 10. **b** Histograms representing survey responses to question 12. **c** Histograms representing survey responses to question 14

In 65% of cases, respondents claimed to use validated screening tools (e.g. MUST, MNA, MST, NRS 2002 [9], other) to identify malnutrition risk in HNC patients undergoing radio(chemo)therapy. Clinicians reported that they would use prophylactic nutritional support in 85% of cases suffering from severe dysphagia during any treatment course; placement of a feeding tube directly into the stomach/jejunum (i.e. PG/PJ) was the first choice of nutritional support in 46% of cases, nasogastric or jejunal feeding tube was used in 36%, while intravenous devices (i.e. cannula introduced in a peripheral vein and reaching the cava vein or cannula inserted in a central vein) were used as last resort in 4–5% of cases (Fig. 1a).

**Fig. 2:**
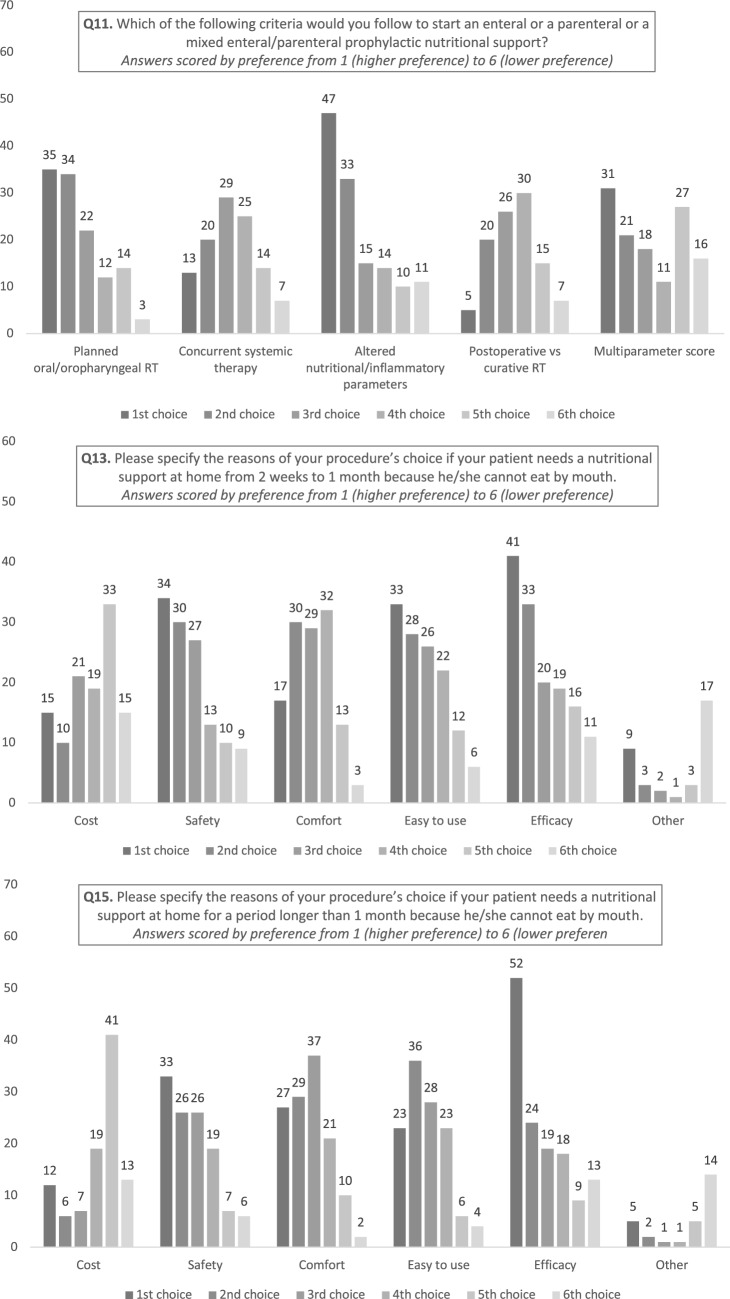
**a** Histograms representing survey responses to question 11. **b** Histograms representing survey responses to question 13. **c** Histograms representing survey responses to question 15

Most respondents reported that they used altered nutritional or inflammatory parameters (i.e. weight loss, Glasgow prognostic score, low phase angle, sarcopenia) before or during therapy (first criteria of choice in 36%, second in 25% of cases), as the principle factor that determined nutritional intervention. The second most adopted criteria was followed by the planned radiation dose on the oral and oropharyngeal mucosa and/or pharyngeal constrictor muscles (first criteria of choice in 29%, second in 28% of cases) as the principle factors that determined nutritional intervention. The setting of RT (23–26%; postoperative vs curative) and the use of concomitant chemotherapy (26–31%) were reported to be less influential in this regard (Fig. 2a).

The choice of prophylactic nutritional intervention differed depending on the duration of the patients’ severe dysphagia. Respondents reported that their first choice would be nasogastric or nasojejunal feeding tube placement in 62% of patients unable to take oral intake for 2 weeks to 1 month (Fig. 1b); however, PG/PJ was preferred (79%) if it was felt that the patient would need nutritional support for a period of longer than 1 month (Fig. 1c).

When considering the approach to prophylactic nutritional support, safety and efficacy were described as the most important variables by 23% and 27.5% respondents, respectively, for dysphagia lasting 2 weeks to 1 month (Fig. 2b) and by 22% and 34%, respectively, for dysphagia lasting longer than 1 month (Fig. 2c). The cost of the procedure was the last criteria of choice in the majority of cases (25% both for period of dysphagia ranging from 2 weeks to 1 month and longer than one month).

Nearly all of the respondents (91%) considered patients' preferences for the nutritional approach to be used. In more than half of the cases (61%), respondents reported that patients’ first choice would be a feeding tube directly entering into the stomach/jejunum, followed by nasogastric or nasojejunal feeding tube in 27% of cases. Clinicians reported that in their opinion patients considered intravenous nutrition as a last choice (18%).

In addition to the clinical-nutritional criteria already evaluated, respondents felt that the patient’s age was also a relevant parameter indicating the need for earlier nutritional support in 70% of cases; the commonest age cut-off among the respondents was 70 years old (39%).

**Fig. 3:**
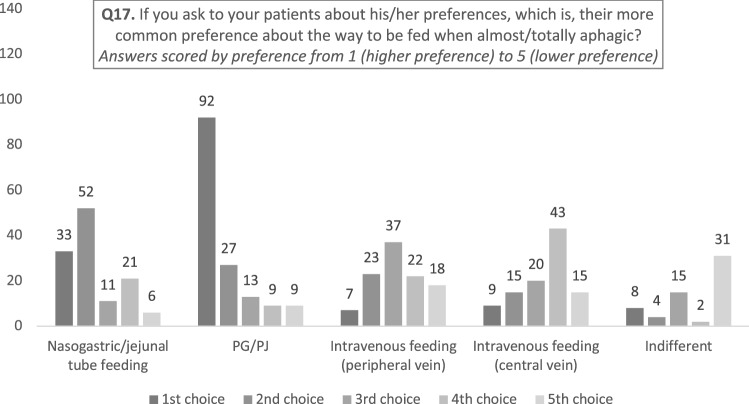
Histogram representing survey responses to question 17

Finally, the reported preferences of patients with regard the route of feeding is demonstrated in Fig. 3.

## Discussion

The survey was designed to define the current practice of nutritional support of HNC patients when receiving oncological therapy. Data collected within this survey represent a snapshot of the current nutritional approach to managing HNC patients in Europe. Notably, the majority of the respondents were from south and middle-Europe countries and from specialised centers (Academic Institutions and Cancer Centres). Therefore, the findings of the questionnaire likely reflect the considered approach adopted by experienced and qualified health care professionals from these regions.

The data clearly show that, regardless of the timing of the recommendation (“prophylactic” nutrition, nutrition required for 2–4 weeks, or expected longer than 1 month), the enteral route to feeding was always preferred. Furthermore, when the predicted duration of nutritional support was relatively short (less than 1 month) the nasal approach was preferred by 50% of medical and radiation oncologists, 54% of surgeons and 64% of nutritionists/dieticians (data not shown). In contrast, insertion of a percutaneous gastric tube was felt to be indicated by the majority (81.0%) of respondents, regardless of their specialty, if nutritional support was warranted for longer than 1 month. Only a small proportion of clinicians felt that the intravenous route should be used as the first for nutritional support.

The prevalent reason (34.0%) for recommending a prophylactic nutritional support was the presence of malnutrition detected according to the standard nutritional parameters and/or presence of inflammatory indexes. It is interesting, however, that clinicians felt that other factors, such as the planned radiation dose on oral and oropharyngeal mucosa and/or pharyngeal constrictor muscles (25.3%) or the result of multiparameter risk scores (22.4%) also played a significant role. The combined use of the latter two parameters, accounting for about half (48.7%) of the indications, may reflect a notably new practice because it underlines the need for a personalised comprehensive risk screening procedure that also includes also non-nutritional variables. The relevance of a comprehensive evaluation of the nutritional risk, including not only the nutritional aspects but also the burden of therapies, was not enough emphasized in the recent European [5] and American [6] guidelines, although some analyses on the risk of surgical complications following major surgery [10–15] underlined this concept. Focusing on HNC area, is noteworthy that prediction of major weight loss was found to be also dependent on non-nutritional parameters such as HNC site and stage [16], the total planning target radiation volume, prescription dose planning target volume and the use of chemotherapy [17]. Mays et al. [18] reported that the following variables in addition to preoperative weight loss and dysphagia were significant and independent predictors of gastric tube placement: preoperative irradiation, supra-cricoid laryngectomy, tracheostomy tube placement, clinical node stage and reconstruction type and developed a predictive model based on these variables. Thus, it appears quite reasonable that the indication for nutritional intervention should be contextualized within a complex decisional process involving the patient’s clinical status, the characteristics of the tumour and the modality of the therapeutic approach.

Clinicians tended to prefer the enteral route for nutritional support because of a perception of improved safety (21.7–22.8% of the answers), better comfort (11.4–17.7% of answers) and easier use (15.5–21.5%). Collectively, these practical considerations dictated the choice of the enteral route in about half of the respondents, in keeping with both the European [5] and the American [6] guidelines.

It is interesting that only 27–34% of respondents scored “efficacy” as the first single criterion for selecting the type of nutritional approach. Questionnaire’s answers cannot discriminate whether the choice of the enteral intervention depends on a major practicability of the enteral route, despite similar efficacy with intravenous rout, or, instead, it reflects the belief of a better efficacy per se of the enteral nutrition. On this point it is important to underline that few metabolic short-term studies [19–22] comparing short-term EN with PN have not found differences between the two routes as regards the potential for nutritional repletion. However, the major complication rate in cancer patients seemed equivalent between the two procedures according to a recent meta-analysis, except for infection rate being higher with PN, which is a critical issue during radio(chemo)therapy [23].

Finally, it is noteworthy that 61% clinicians felt that aphagic patients would prefer PG/PJ as the modality of choice. This percentage should be contextualized within the three different nutritional intervention scenarios we have considered (prophylactic, 2–4 weeks, > 1 month) where PG/PJ were recommended in 46%, 29% and 81% of cases.

In conclusion, the analysis of the results of this questionnaire can offer some hints of practical interest, as well as suggestions for future research.

First, it appears there is an adequate awareness of the relevance of nutritional interventions among clinicians involved in the care of patients with HNC, albeit with different positions regarding some practical choices.

There remain some unresolved issues which mainly concern the option for the prophylactic use of PG/PJ as a short-term nutritional intervention. In fact, preferences for nasogastric/jejunal tube feeding were reported in about half of the respondents, the remaining part preferring other solutions.

A few small randomized trials showed that nasogastric tube feeding [24, 25] or early PG [26, 27] maintained weight or prevented weight loss better than optimal oral nutrition alone. Corry et al. [28] showed a benefit for weight gain in PG versus nasogastric tube feeding patients only at 6 weeks, but no difference at 6 months, with overall QoL scores and complication rates being similar. Meta-analyses [29] reported no significant differences in the overall complication rate between nasogastric tube feeding and PG even if tube dislodgement was more frequent in nasogastric tube feeding and late dysphagia in PG patients [30]. Discrepancies among the results of trials may depend on the heterogeneity of series as regards treatment and disease characteristics, comorbidities, the nutritional regimens of the control groups as well as a lack of correct stratification of the patients by risk scores for malnutrition and hypophagia [16–18].

As regards the effects of prophylactic PG versus PG placement when required, a large study [31] showed no difference between the two procedures on body weight and survival and a similar result was noted in a systematic review [32]. However, a more recent review [33] reported that the prophylactic PG strategy was associated with decreased malnutrition during treatment and improved QoL at 6 months, even if it was associated with higher rates of long-term gastrostomy dependence. Timing of PG placement was not associated with improvement in tumour control or overall survival.

Another field of discussion relates to oral supplements, which was not addressed in this survey. Recently, a randomized controlled trial [34] has shown that in HNC patients undergoing RT or RT plus systemic treatment, and receiving nutritional counselling, the use of oral nutritional supplements resulted in better weight maintenance, increased protein-calorie intake, improved QoL and better anti-cancer treatment tolerance.

In conclusion, administration of oral nutritional supplements, especially those with protein- and ω-3- enriched formulas, remains underutilized and certainly represents an open issue for further studies.

A conservative approach which would appear underutilized is the combined oral-parenteral feeding in patients with mild or short-term dysphagia since a recent investigation on a mixed series of patients, including also HNC, showed that an early 7-day supplemental parenteral nutrition improved body composition and muscle strength in hypophagic cancer patients [8].

A final interesting point is the preference of the route by the almost aphagic patients: 90% of the responders reported their patients would opt for a PG/PJ (61.7%) or a nasogastric feeding (22.1%), the intravenous route accounting for 10.7% only, a finding just opposite to the inquiry of Scolapio et al. [35] who reported in a heterogeneous population of oncological patients that most prefer intravenous to nasal gastric feeding.

There are some limitations to this study: first, answers came from a selected group of clinicians who are strongly involved in HN cancer and this hampers any generalization of our conclusions. Second, questions had a practical and sometimes mutually exclusive approach preventing more articulated answers and finally some discrepancies in the number of respondents to different questions could make extrapolations or correlations of data from different questions somewhat arbitrary.

There are also some points of strength: answers from different countries and/or specialists showed a relatively homogeneous and consistent pattern about indications, and pointed out the presence of still grey areas for some indications which appear to rely more on an usual practice than on evidence and thus provided some hints for future investigations.

## Data Availability

All data generated or analysed during this study are included in this published article.
